# Supplementary figures and images for: Comparison of traditional methods versus SAFEcount for filling prescriptions: A pilot study of an innovative pill counting solution in eSwatini
Source: PLoS One. 2019 Dec 4;14(12):e0224323. doi: 10.1371/journal.pone.0224323 (PMC6892485; doi:10.1371/journal.pone.0224323)

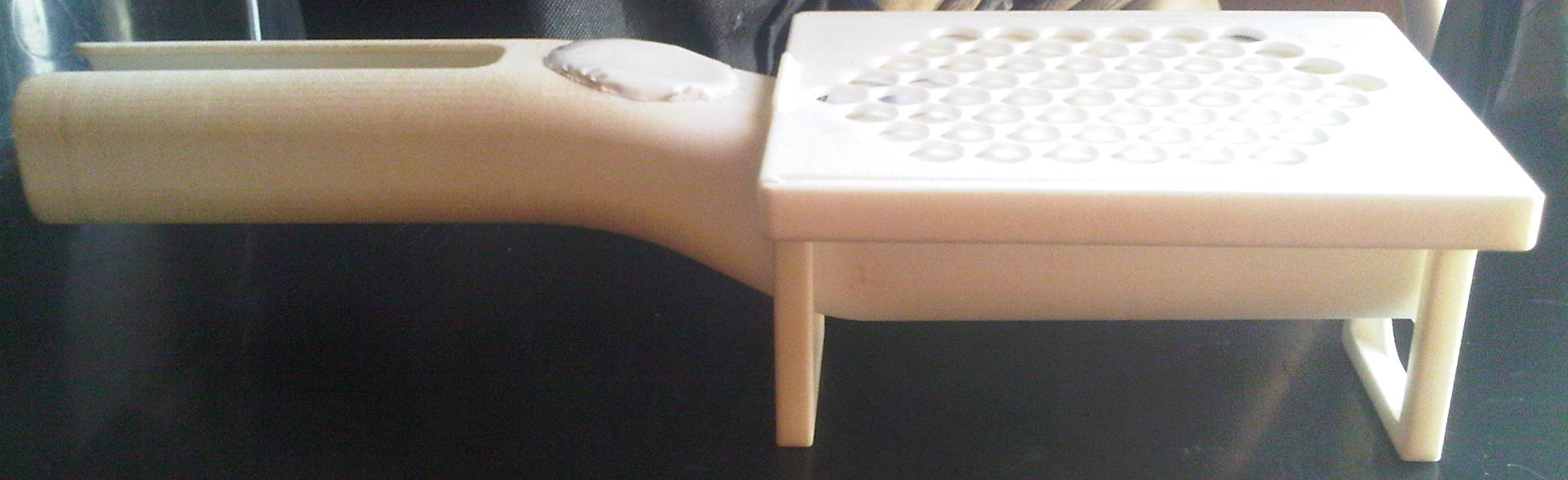

Supplement: S1 Photograph — (TIF) [file pone.0224323.s001.tif]

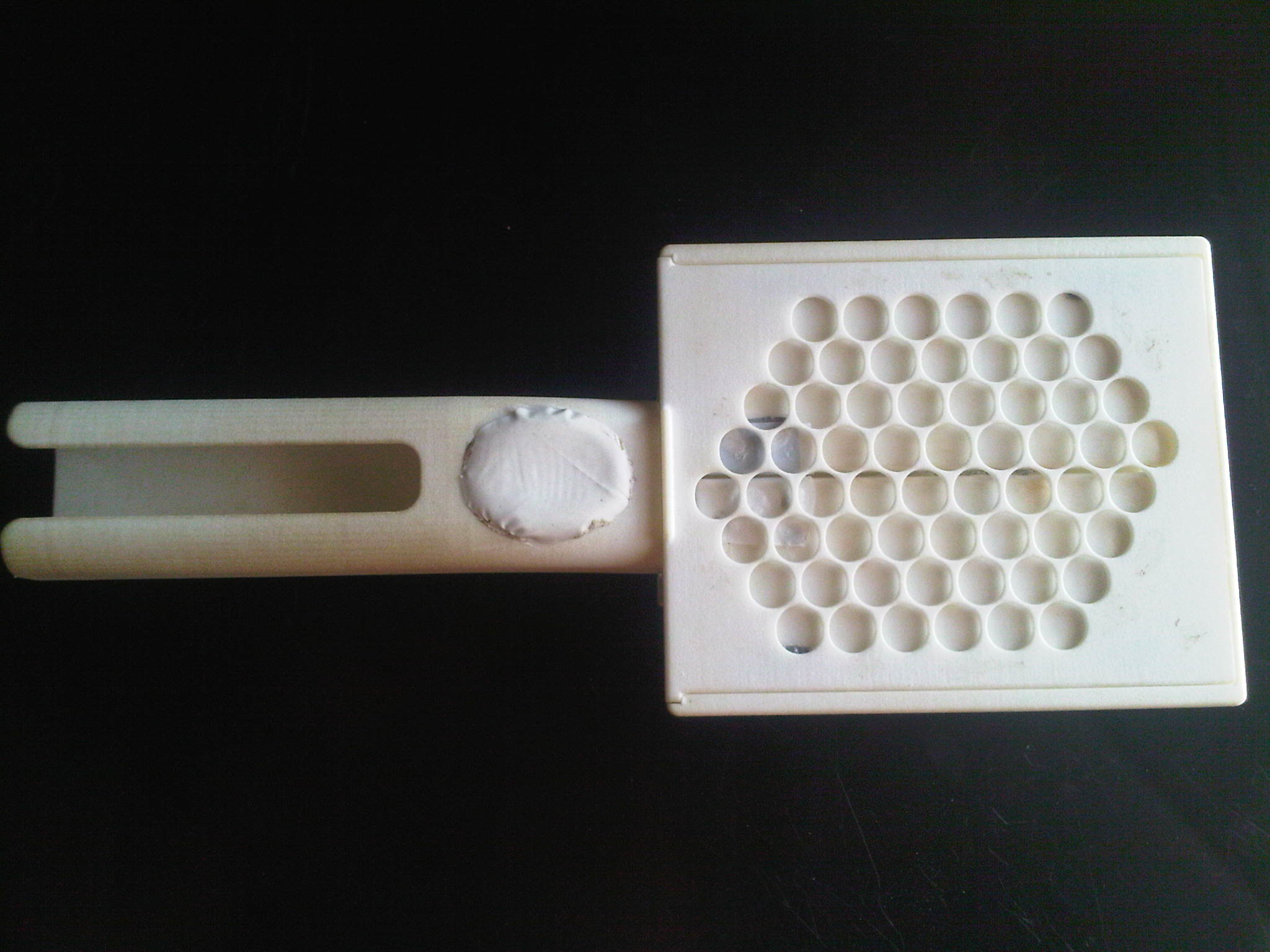

Supplement: S2 Photograph — (TIF) [file pone.0224323.s002.tif]

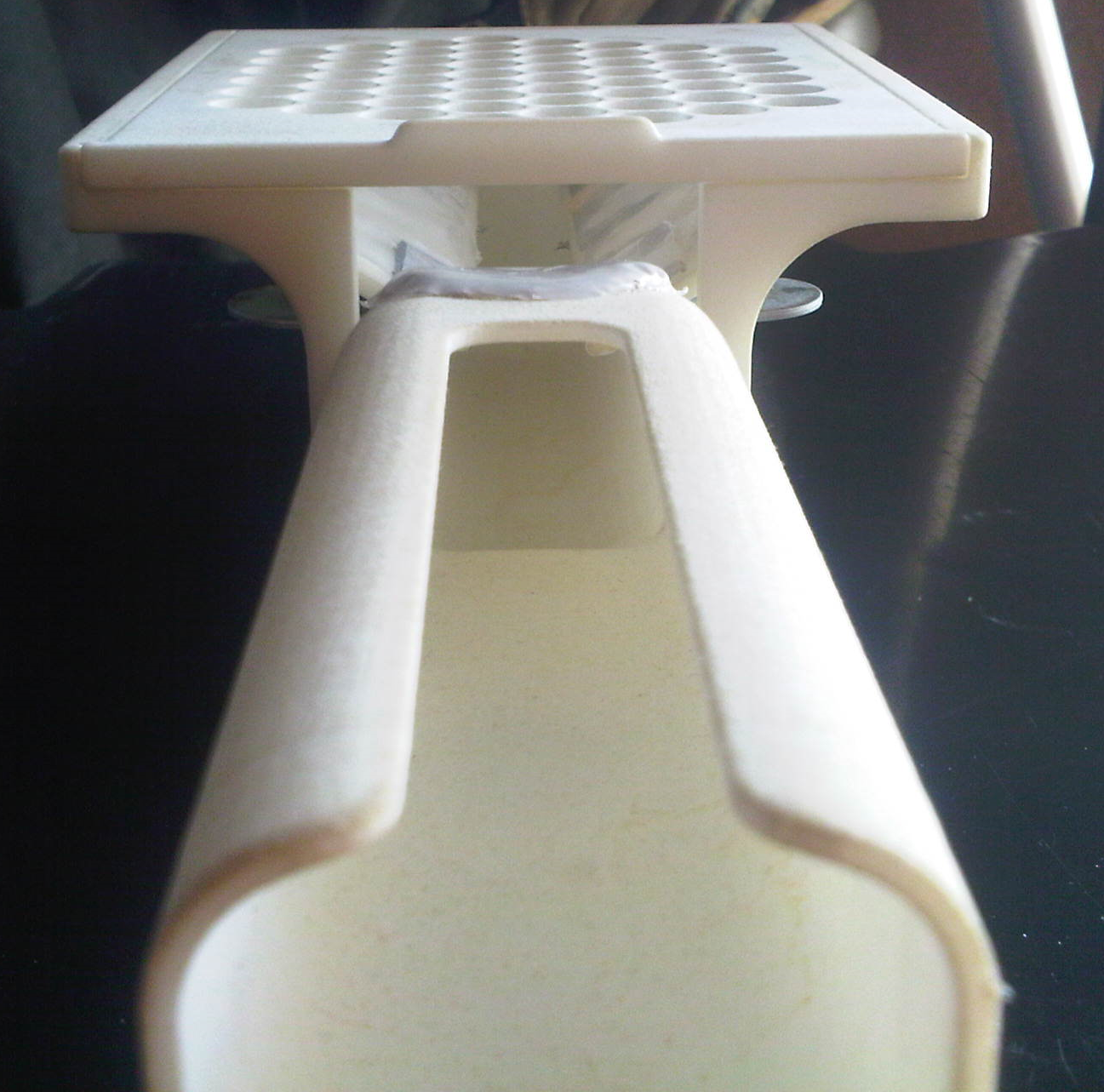

Supplement: S3 Photograph — (TIF) [file pone.0224323.s003.tif]
